# Alginate-based hydrogel platform embedding silver nanoparticles and cisplatin: characterization of the synergistic effect on a breast cancer cell line

**DOI:** 10.3389/fmolb.2023.1242838

**Published:** 2023-10-23

**Authors:** Shaimaa Maher, Haitham Kalil, Guiming Liu, Khalid Sossey-Alaoui, Mekki Bayachou

**Affiliations:** ^1^ Chemistry Department, Cleveland State University, Cleveland, OH, United States; ^2^ Department of Chemistry, Faculty of Science, Suez Canal University, Ismailia, Egypt; ^3^ Department of Surgery, MetroHealth Medical Center, Case Western Reserve University, Cleveland, OH, United States; ^4^ Department of Medicine, Case Western Reserve University, Cleveland, OH, United States; ^5^ Metro Health Medical Center, Cleveland, OH, United States; ^6^ Department of Inflammation and Immunity, Cleveland Clinic, Lerner Research Institute, Cleveland, OH, United States

**Keywords:** silver nanoparticles, cisplatin, breast cancer, nanocomplex, alginate

## Abstract

**Introduction:** Breast cancer is a significant cause of mortality in women globally, and current treatment approaches face challenges due to side effects and drug resistance. Nanotechnology offers promising solutions by enabling targeted drug delivery and minimizing toxicity to normal tissues.

**Methods:** In this study, we developed a composite platform called (Alg-AgNPs-CisPt), consisting of silver nanoparticles coated with an alginate hydrogel embedding cisplatin. We examined the effectiveness of this nanocomplex in induce synergistic cytotoxic effects on breast cancer cells.

**Results and Discussion:** Characterization using various analytical techniques confirmed the composition of the nanocomplex and the distribution of its components. Cytotoxicity assays and apoptosis analysis demonstrated that the nanocomplex exhibited greater efficacy against breast cancer cells compared to AgNPs or cisplatin as standalone treatments. Moreover, the nanocomplex was found to enhance intracellular reactive oxygen species levels, further validating its efficacy. The synergistic action of the nanocomplex constituents offers potential advantages in reducing side effects associated with higher doses of cisplatin as a standalone treatment. Overall, this study highlights the potential of the (Alg-AgNPs-CisPt) nanocomplex as a promising platform embedding components with synergistic action against breast cancer cells.

## 1 Introduction

Breast cancer is second in cancer-related mortality among women, behind lung cancer (National breast cancer coalition, 2022). Since 1975, American women’s lifetime risk of invasive breast cancer has increased (Wang et al., 2022). In 2022, the American Cancer Society anticipated 287,850 invasive breast cancer cases in women, 2,710 in males, and 51,400 ductal carcinoma *in situ* (DCIS) cases in women (National breast cancer coalition, 2022). Younger women are significantly less likely to develop invasive breast cancer than older women (Hankey et al., 1994; Shannon and Smith, 2003). It affects an average of one in eight women in 2022 (Alphandéry, 2014). In recent years, breast cancer mortality reduction has slowed down. The breast cancer morality was estimated at 43,250 for women and 530 for men in 2022 alone (National breast cancer coalition, 2022). Among ways to lower the likelihood of developing breast cancer is to control risk factors such as high saturated fat diet, obesity, excessive alcohol consumption, exposure to ionizing radiation, oral contraceptive use, and hormone replacement therapy (Matsen and Neumayer, 2013; Tinoco et al., 2013). Performing periodic diagnostic test offers the possibility of early detection (Joy et al., 2005). Breast cancer treatments, typically include conservative surgery, which involves removing the tumor without removing the entire breast and is usually referred to as a lumpectomy or partial mastectomy (Alphandéry, 2014; Silverstein et al., 2014), and “non-conservative” surgery, which involves the removal of the entire breast and is usually referred to as a total mastectomy (Tasmuth et al., 1996). Radiation therapy is also regularly recommended for patients who have previously undergone conservative surgery or who have metastases that appear at an advanced stage of breast cancer (Boice Jr et al., 1992; Haussmann et al., 2020). Radiation destroys tumors through the ionization of biological tissues, which generates free radicals that interact with cells and cellular constituents including and DNA, resulting in the death of tumor cells. Despite the advancements in radiation therapy, side effects of toxicity the possibility of destroying healthy tissues along with breast cancer cells still exists. Another treatment regimen includes chemotherapy that is typically delivered intravenously to have a systemic effect (Fraguas-Sánchez et al., 2019). The limitations of chemotherapy are caused by the nonspecific targeting of tumor cells (Cao et al., 2018), which leads to undesirable effects such as hair loss and weight gain in women (Khan et al., 2019). Multi-drug resistance as a consequence of recurrent medication administration throughout a chemotherapy treatment cycle has been recognized as the main reason for chemotherapy failure (Bukowski et al., 2020). Additionally, traditional chemotherapy is less effective when anticancer drugs are distributed systemically throughout the body and do not accumulate preferentially in the tumor (Bathara et al., 2020). The quality of life for cancer patients may be negatively impacted by unfavorable concentrations of anticancer medicines in healthy tissues. Hormonal treatment reduces estrogen and progesterone, which cause hormone-sensitive tumors in 80% of breast cancer patients (Abel et al., 2022). Hormone therapy can cause side effects such as menopause, osteoporosis, and hair loss (Hankey et al., 1994; Wang et al., 2022). Targeted drug therapy employs drugs that target proteins on breast cancer cells that promote their growth, spread, and survival (Wang et al., 2019). Targeted drugs work to destroy or slow down the growth of cancer cells (Abbas and Rehman, 2018).

In this study, we attempt to address the previously mentioned issues with traditional breast cancer treatment using a nanotechnology platform that hosts a synergistic combination therapy. Nanotechnology advancements using materials with dimensions ranging from 1 to 100 nm for breast cancer treatment, make chemotherapy more efficient, less toxic, and more successful (Reddy, 2020). Nanotechnology provides strategies that target and address the limitations of traditional chemotherapies, and offers significant benefits for cancer patients (Li et al., 2021). Among the various metallic nanoparticles (NPs), silver (Ag) nanoparticles are rapidly gaining attention due to their lower reactivity and promising therapeutic applications as an anticancer agents (Ong et al., 2013). In addition to their lower reactivity, other advantages of using AgNPs as anticancer therapeutic agents include their ease of preparation, easy access to different sizes and shapes, and their physical stability that allow them to remain monodispersed for longer periods of time. Another advantage is their synergistic photothermal and chemo responses and well-understood surface plasmon resonance artifacts. The possibility to host drug loads with synergistic and combinatorial pharmacological effects is another advantage of this potential therapeutic platform (Zhang et al., 2016). Sangiliyandi Gurunathan et al. have successfully developed stable AgNPs with a diameter of 20 nm using the bacterium B. funiculus. They highlighted the potential cytotoxic effect of biologically synthesized AgNP on MDA-MB-231 cells. Their data suggest that oxidative stress contributes to nanoparticle cytotoxicity. They demonstrated that AgNPs possess antiproliferative activity by inducing apoptosis in the MDA-MB-231 breast cancer cell line, indicating that AgNPs may be a potential therapy for breast cancer in humans (Gurunathan et al., 2013). The bioavailability, biocompatibility, and stability of nanoparticles can be enhanced by modifying their surfaces with an appropriate coating. Numerous studies have confirmed the potential utility of alginate-based platforms as effective drug delivery vehicles for cancer-specific treatments. Sodium alginate (SA) is one of the most common naturally occurring polysaccharides (Mukarram et al., 2021). Alginates are formed by Laminaria hyperborea, Macrocystis pyrifera, and other brown algae that grow in coastal waters all over the world (Pereira et al., 2011). It is made of *ß*-D-mannuronic acid (M block) and α-L-guluronic acid (G block) units linked by *ß*-1,4- glycosidic bond. Alginate has great biodegradability, low toxicity, chemical functionality, crosslinking ability, high water content, soft consistency, pH sensitivity, as well as biocompatibility, which makes it a good candidate as a drug carrier to deliver low molecular weight drugs and macromolecules like proteins and genes. Biocompatibility of alginate is a result of hydrophilicity, chain migration, and water absorption.

Combination therapy, which refers to the concurrent administration of multiple medications, has become prevalent in clinical oncology in recent years (Pegram and Slamon, 1999; Núñez et al., 2016; Lee and Djamgoz, 2018). The main benefit of combination therapy is that different treatments can work together to create synergistic effects that are stronger than the effects of using the same treatments individually (Pauzi et al., 2016). Also, changing the doses of the individual treatments in combination therapy could help reduce side effects caused by high doses, which helps reduce the problem of drug resistance. Cis-diamminedichloroplatinum (CDDP), also simply known as cisplatin, is one of the highly effective anti-cancer agents presently used in medical care, with prominent action against a range of cancers, including breast (Sledge Jr et al., 1988), testicular (de Vries et al., 2020), and ovarian cancers (Fahrenholtz et al., 2017). Cisplatin interacts with DNA and hinders the transcription and replication mechanisms (Marchán et al., 2001). However, cisplatin treatment is associated with a number of undesirable side effects, including renal damage, bone marrow suppression, ototoxicity, allergy reactions, peripheral neuropathies, and hypomagnesemia (Von Hoff et al., 1979; Gregg et al., 1992). In addition, the development of resistance mechanisms in cancer cells limits the clinical application of cisplatin (Pogribny et al., 2010; Galluzzi et al., 2012). Accordingly, combining cisplatin with other anti-cancer drugs with a distinct method of action might suppress cisplatin-resistant cancer cell proliferation. This strategy might lessen adverse effects while sustaining or boosting therapy effectiveness. (Pegram and Slamon, 1999; Pauzi et al., 2016; Fahrenholtz et al., 2017). The goal of this study is to use both nanotechnology (silver nanoparticles or AgNPs as a platform) and combination therapy (by combining silver nanoparticles and cisplatin on a single platform with and without alginate) to study the effect on MDA-MB-231 breast cancer cell line. The idea is to investigate if there are cumulative effects that increase the synergy of the constituents on the composite nanoparticle platform, which ultimately would overcome the undesirable side effects of standard breast cancer treatments.

## 2 Materials and methods

### 2.1 Materials

Sodium alginate with low viscosity was obtained from MP Biomedicals. Sodium borohydride, Acridine Orange (AO), Propidium Iodide (PI) stain, and Penicillin/Streptomycin (P/S) were purchased from Thermo Fisher Scientific. Silver nitrate (AgNO_3_), 2′, 7′-Dichlorofluorescin Diacetate (DCFH-DA), cisplatin, and 3–(4,5-dimethylthiazol-2-yl)-2,5-diphenyltetrazolium bromide (MTT) were obtained from Sigma Aldrich. MDA-MB-231 cells were purchased from the American Type Culture Collection (ATCC). Dulbecco’s Modified Eagle Medium (DMEM) was bought from Cytiva HyClone Laboratories. Sterile fetal bovine serum (FBS) was supplied from Hyclone GE Healthcare. The mixture of trypsin-DTA and mouse antibody against α-tubulin was supplied by Millipore Sigma. FITC Annexin V Apoptosis Detection Kit I (BD Biosciences, Heidelberg, Germany). Rabbit antibodies against Bax and *Bcl-2* were purchased from Cell Signaling Technology. All sterile plasticware for tissue culture was sourced from Corning. All the chemicals used were of analytical quality, and ultrapure water was used throughout.

### 2.2 Synthesis of alginate coloaded with silver nanoparticle (AgNPs) and embedding with cisplatin

Silver nitrate (AgNO_3_) was reduced with sodium borohydride (NaBH_4_) to produce AgNPs as described in previous publications with some modifications (Keshavarz et al., 2018; Mirrahimi et al., 2019; Mirrahimi et al., 2020). A mixture of silver alginic acid sodium salt was prepared by adding 17 mL of 14.7 mM solution of AgNO_3_ drop by drop into 50 mL of freshly prepared sodium alginate solution (0.3%). The mixture was then transferred to round bottom flask on ice. Thereafter, a fresh 10 mL solution of 10 mM sodium borohydride was added to the mixture under stirring. The mixture turned yellow suggesting that silver ions were being reduced, and silver nanoparticles (AgNPs) were being formed. For drug loading, a solution of cisplatin with a concentration of 100 ppm was added dropwise into the AgNPs/alginate mixture at room temperature under fast stirring to provide the Alg-AgNPs-CisPt nanocomplex.

### 2.3 Characterization of the synthesized nanocomplex

The Alg-AgNPs-CisPt nanocomplex was characterized using an UV-vis (Schimadzu 1800). Scanning electron microscopy and Energy-dispersive X-ray Spectroscopy (INSPECT F50, FEI) were used to examine the surface morphology and elemental analysis of the nanocomplex. The shape and size distribution of the nanocomplex’s particles were analyzed using transmission electron microscopy (FEI Tecnai T12). ImageJ software (Rasband et al., 1997), which is freely available, was used for image analysis. Zeta potential measurements were measured with the Malvern Zetasizer Nano ZS-90 instrument.

### 2.4 *In vitro* cytotoxicity using the MTT assay

The cytotoxicity of the synthesized nanocomplex against MDA-MB-231 cancer cells was assessed using the MTT assay (Mukherjee et al., 2012). After a few passages, MDA-MB-231 cells were seeded at a density of 5*10^4^/mL cells per well in 96-well plates and allowed to adhere overnight. The cells were then incubated for 24 h with the nanocomplex containing silver nanoparticles at concentrations ranging from 0 to 20 ug/mL and cisplatin ranging from 0 to 25 ppm. In addition, the cytotoxicity of AgNPs alone and free cisplatin at concentrations comparable to those encapsulated in the synthesized nanocomplex was evaluated. The concentrations based on AgNPs were: 0, 1.25, 2.5, 5, 10, and 20 μg/mL and the concentrations based on cisplatin were: 0, 1.56, 3.125, 6.25, 12.5, and 25 ppm. After the set incubation time is reached, the cells were washed twice with PBS buffer to get rid of any remaining free nanoparticles in the culture media before resuspension in a new medium. Then, 20 uL of MTT reagent (5 mg/mL in PBS) was added to the wells, and the cells were incubated for an additional 4 h. The medium containing MTT reagents was then removed and was replaced with 100 µL DMSO to dissolve the formazan crystals. The plates were analyzed after 15 min of shaking in absence of light at room temperature. The absorbance of the dissolved formazan was measured at 570 nm using a reference wavelength of 630 nm on an ELISA reader. The following formula (Gurunathan et al., 2013; Remya et al., 2015; Prasannaraj and Venkatachalam, 2017) was used to determine the proportion of live cells based on their optical density (OD) value:

Percentage of cell viability = (OD value of experimental treated sample/OD value of experimental untreated sample as control) *100.

Half-maximal inhibitory concentration (IC50) Calculation:

Cytotoxicity percentage obtained from the MTT assays for AgNPs, Nanocomplex and Cisplatin are shown in Figure 8A. For the curve fitting, the concentrations were expressed in a logarithmic scale and the IC50 was calculated through the four-parameter logistic (4 PL) regression. The equation that described the curves is as follows y = b + (a - b)/(1 + (x/c)^d).

Where:

• y represents the cytotoxicity percentage.
• x represents the concentration of the nanocomplex or its standalone components
• The four parameters are as follows:
  a) the minimum response that can be obtained.
  b) the maximum response that can be obtained.
  c) the inhibitory concentration at 50% response (IC50).
  d) Hill’s slope of the curve, which is related to the steepness of the curve at point c.

### 2.5 *In vitro* staining by acridine orange/propidium iodide

MDA-MB-231 Cells were seeded in a 6-well plate to a density of 4*10^5^/mL cells per well and incubated under 5% CO_2_ at 37°C until 90% confluence was reached in the culture medium (90% Dulbecco’s Modified Eagle Medium high glucose concentration, 10% fetal bovine serum, 100 units/mL penicillin, and 0.01 mg/mL streptomycin solution). The cells were then incubated for 24 h with two different concentrations of synthesized nanocomplex containing silver nanoparticles (5 and 10 ug/mL) and Cisplatin (6.25 and 12.5 ppm). In addition, the cytotoxicity of AgNPs alone and free cisplatin at concentrations comparable to those encapsulated in the synthesized nanocomplex was evaluated (concentration based on AgNPs: 5 and 10 ug/mL; and concentration based on cisplatin: 6.25, and 12.5 ppm). Alternatively, the untreated cultured cells served as the control group. After incubation, the cells were washed twice with PBS to remove residual free nanoparticles from the culture medium. After each incubation period, the cells were stained with acridine orange (AO) and propidium iodide (PI) for 15 min at 37°C and 5% CO_2_, and then the medium was replaced with a fresh one (Azizi et al., 2017; Hajebi et al., 2019; Zhang et al., 2019). Labeled cells were analyzed using fluorescence microscopy. For each sample, at least five randomly chosen images were counted in addition to the control. ImageJ was used to merge live and dead cells.

### 2.6 *In vitro* apoptosis/necrosis assay using Annexin V-PI assay

The nanocomplex-induced apoptosis of MDA-MB-231 cells was quantified by flow cytometric analysis using Annexin and PI stains. In six-well plates, 4 * 10^5^/mL MDA-MB-231 cells were seeded and incubated at 37°C until 90% confluence. The cells were then incubated for an additional 24 h with two different concentrations of synthesized nanocomplex containing silver nanoparticles (5 and 10 ug/mL) and Cisplatin (6.25 and 12.5 ppm). In addition, the apoptosis activity of AgNPs alone and free cisplatin at comparable concentrations to those encapsulated in the synthesized nanocomplex (i.e., concentration based on AgNPs: 5 and 10 μg/mL and concentration based on cisplatin: 6.25 and 12.5 ppm) was evaluated. Untreated cultured cells served as the control group. The manufacturer’s protocol was followed to stain treated and untreated cells (BD Biosciences, Heidelberg, Germany). Briefly, after 24 h of incubation, the cells were washed with PBS, trypsinized, centrifuged, collected, resuspended in 100 µL of Annexin V binding buffer solution (1X), and mixed slowly. Then, 5 µL of FITC Annexin V and 5 µL of propidium iodide were added, vortexed gently, and incubated at room temperature in the dark for 15 min. Finally, 400 µL of 1 X binding buffer was added to each tube, and analyzed within 1 h using flow cytometry (Prakash et al., 2013; Delalat et al., 2022).

### 2.7 Western blot analysis

Immunoblotting analyses were conducted as previously described with some modification (Ou et al., 2014; Shebaby et al., 2014; Rana et al., 2021). MDA-MB-231 cells were seeded at a density of 4 × 10^6^/mL per 10-cm Petri dish, and incubated at 37°C with 5% CO_2_ until 90% confluence was achieved. The cells were then treated for 2 h with silver nanoparticles, nanocomplex, and free cisplatin. In addition, untreated cells were used as a control. Prior to lysis, adherent and non-adherent MDA-MB-231 cells were collected on ice and rinsed twice with ice-cold PBS. The adhering cells were lysed with an ice-cold RIPA lysis solution including protease and phosphatase inhibitors by incubation for 30 min on ice. The lysate was transferred to prechilled 1.5-mL microcentrifuge tubes. The samples were then centrifuged for 20 min at 4°C at a speed of 15,000 rpm. The supernatant was transferred to a 1.5-mL microcentrifuge tube, while the pellet was discarded. The Bio-Rad DC Protein Assay was used to measure how much protein the sample contained. Each lysate was denatured in SDS-Polyacrylamide Gel Electrophoresis (SDS-PAGE) 4x sample buffer and *ß*-mercaptoethanol for 5 min at 95°C. The proteins were then loaded onto a 4%–20% sodium dodecyl sulfate polyacrylamide gel (SDS-PAGE) and transferred to a PVDF membrane. The membrane was incubated for 1 h at room temperature in a blocking solution (5% BSA) to avoid non-specific binding. To find the expression of proapoptotic and antiapoptotic proteins, membranes were probed with primary antibodies against Bax, Bcl-2, and α-Tubulin and left at 4°C overnight. The membranes were then washed with TBST 3 times for 5 min each and left at room temperature for an hour with an appropriate HRP-coupled secondary antibody. The membrane was then washed with Tris-buffered saline containing 0.1% Tween 20 (TBST) four times for 5 min each. The signals were developed using a Pierce ECL Western blot chemiluminescence detection kit before being examined and analyzed with the Chemi-Doc MP Imaging System. ImageJ was used to quantify signals according to the criteria outlined in the ImageJ user guide.

### 2.8 ROS detection by 2′,7′-Dichloro dihydro fluorescein diacetate (DCFH-DA) assay using flow cytometry

MDA-MB-231 Cells were seeded at a density of 4*10^5^/mL per well in a 6-well plate and incubated under 5% CO_2_ at 37°C until 90% confluence was reached in the culture medium (90 percent Dulbecco’s Modified Eagle’s Medium), as mentioned earlier. The cells were then incubated with two different concentrations of synthesized nanocomplex based on silver nanoparticles (5 and 10 ug/mL) and based on cisplatin (6.25 and 12.5 ppm) for 24 h. In addition, measurements of ROS in experiments with AgNPs alone and free cisplatin at comparable concentrations to those encapsulated in the synthesized nanocomplex were also evaluated. Untreated cultured cells served as the negative control (−ve), while cells treated with 0.05% and 0.1% H_2_O_2_ for 30 min prior to staining served as the positive control (+ve). After incubation, any leftover free nanoparticles were washed away using two rounds of washing with phosphate-buffered saline solution. The cells were further incubated for 30 min at 37°C with 5% CO_2_ in 10 µM of freshly prepared prewarmed loading buffer (PBS) containing the probe (DCF-DA). The loading buffer was washed twice with PBS and the cells were harvested with trypsin, resuspended in fresh PBS, and analyzed with flow cytometry within 1 h (Lee et al., 2014; Gurunathan et al., 2015).

### 2.9 ROS detection by 2′,7′ Dichloro dihydro fluorescein diacetate (DCFH-DA) assay using fluorescence microscopy

The procedure was the same as described in Section 2.8, except that after staining the cells with the probe, the sample was washed twice, and then 1 mL of PBS as added to each well and examined under a fluorescence microscope (Chang et al., 2017).

### 2.10 Statistical analysis

All data are reported as mean ± standard deviation (SD). The data were analyzed using Origin 2020b software to generate graphs, while GraphPad Prism 9 software was used to analyze the MTT assay. To assess and compare cell viability outcomes between the AgNPs, Nanocomplex, and Cisplatin groups, we conducted a statistical analysis. First, we performed an Analysis of Variance (ANOVA) test to examine overall differences among the groups. Subsequently, to identify specific pairwise differences, a multiple comparison Tukey’s *post hoc* test was applied. The IC50, which represents the concentration of a substance required to inhibit cell growth by 50%, was calculated for each group using the four-parameter logistic (4 PL) regression method. A 95% confidence interval was calculated to provide a measure of the uncertainty around the estimated IC50 values. Throughout the statistical analysis, a significance level of *p* < 0.05 was used to determine statistical significance.

## 3 Results

### 3.1 Characterization of the synthesized nanocomplex

**FIGURE 1:**
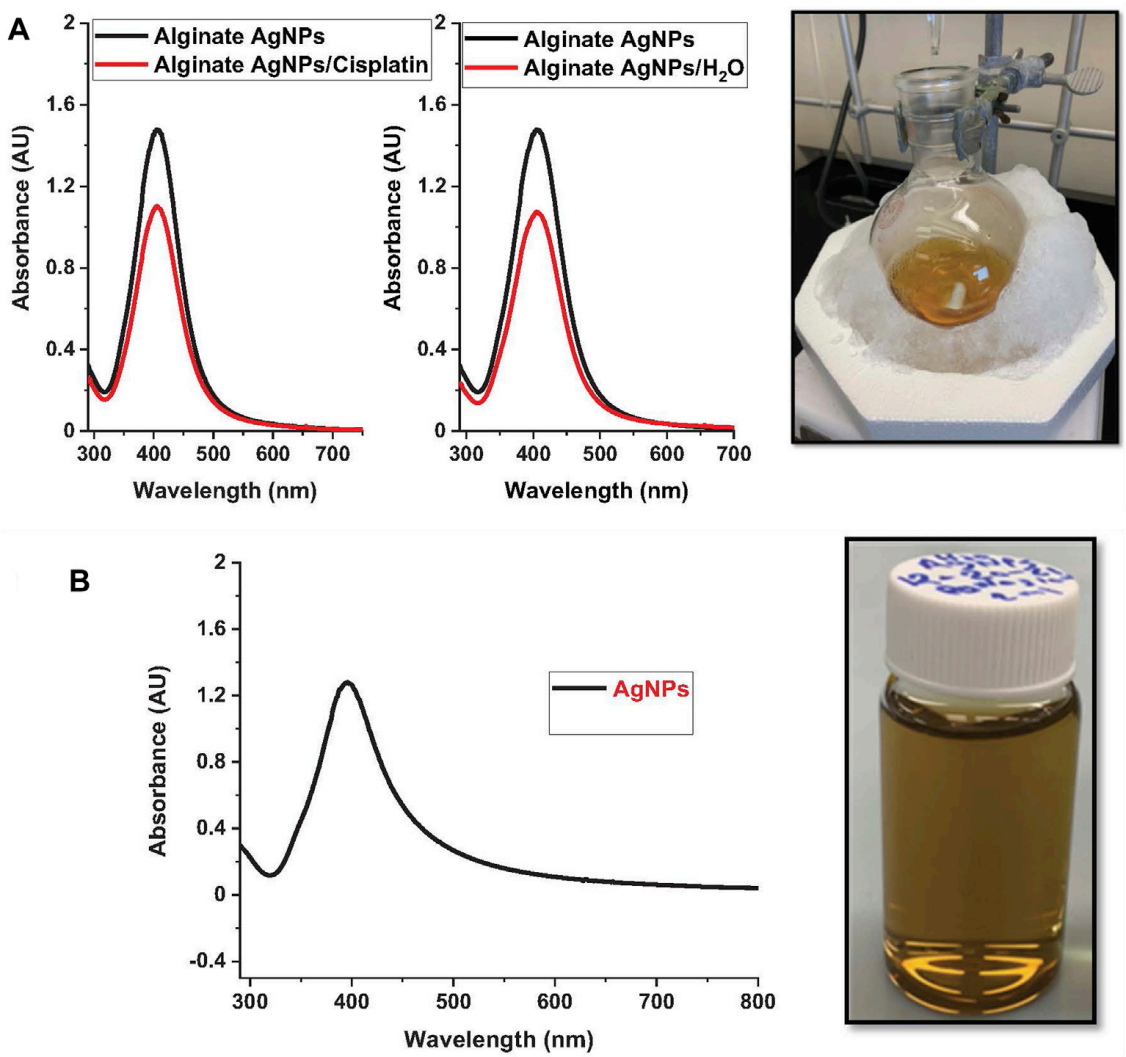
Nanoparticle synthesis. The figure showcases the nanoparticle synthesis process using the protocol described and their respective UV-vis spectra. On the left, nanoparticle suspensions of: **(A)**. Alginate silver nanoparticles (Alg-AgNPs). **(B)**. Silver nanoparticles (AgNPs).

UV–vis spectroscopy is an adequate technique for characterizing AgNPs due to the excitation of surface plasmon resonance band in the AgNPs. It identifies the plasmon band’s evolution as a function of Ag concentration. Using UV–vis spectroscopy, the optical properties of alginate loaded with silver nanoparticles and silver nanoparticles without alginate were measured, Figure 1. After adding sodium borohydride, the color of the alginate silver nitrate solution changed visibly to a yellowish color. This is consistent with previous reports (Chang et al., 2017). All spectra demonstrate an absorption band between 400 and 410 nm, which is the typical plasmon resonance band of silver nanoparticles. To determine the effect of cisplatin on AgNPs, we repeated the UV-Vis measurements after adding cisplatin and found that cisplatin decreases the peak of AgNPs, but the decrease was due to dilution only and not to any other physicochemical process. This was confirmed by replacing the same volume of cisplatin with pure water, which gave the same band intensity decrease.

**FIGURE 2:**
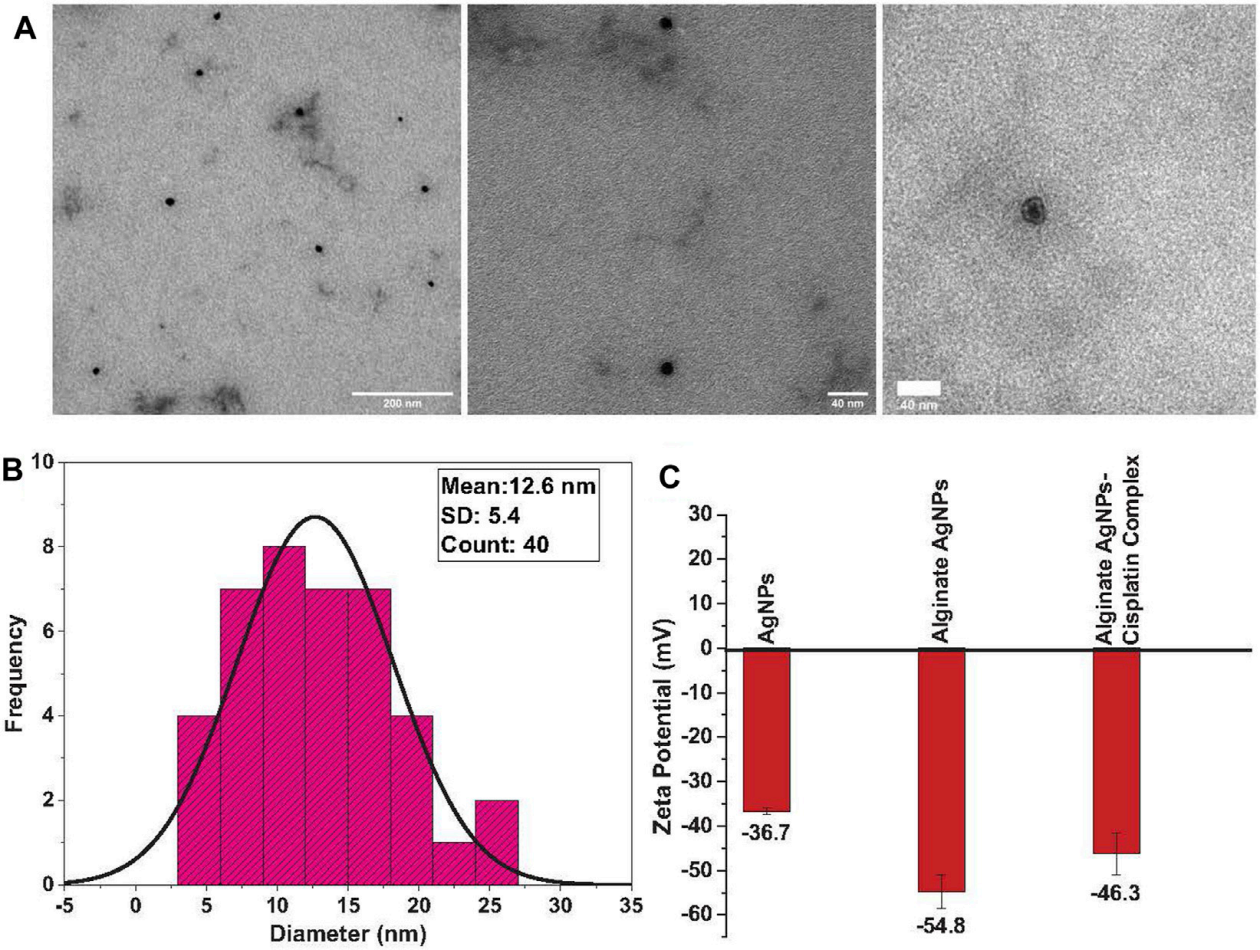
TEM analysis of synthesized alginate AgNPs: **(A)**. Transmission Electron Microscope (TEM) images of the synthesized Alginate AgNPs reveal their spherical shape and entrapment within the alginate hydrogel, demonstrating a monodispersed distribution. **(B)**. Size distribution analysis of TEM-derived Alginate AgNPs shows a range between 4 and 30 nm, with an average size of 12.6 nm. **(C)**. Zeta potential measurements of AgNPs, alginate-coated AgNPs, and the synthesized nanocomplex are presented as mean ± standard deviation (SD).

**FIGURE 3:**
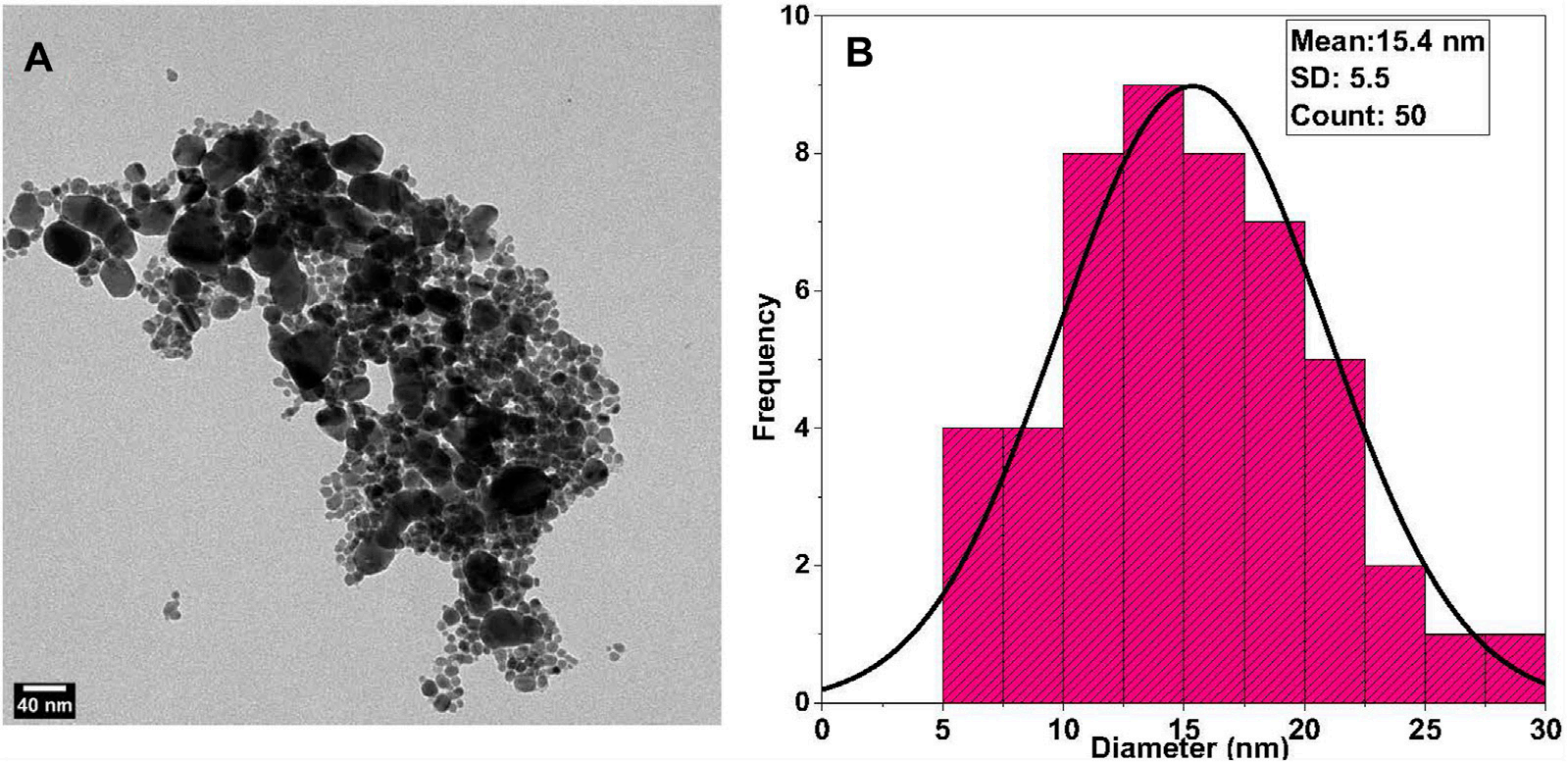
TEM Analysis of Synthesized AgNPs: **(A)** Transmission Electron Microscope (TEM) images of the synthesized AgNPs without alginate reveal a semicircular shape with evident agglomeration. **(B)** Size distribution analysis of TEM-derived AgNPs shows an average size of 15.4 nm.

The particle size of silver nanoparticles ranges from 4 to 30 nm, with an average size of 12.6 nm and a 5.4-nm standard deviation for alginate AgNPs, Figure 2B. AgNPs in absence of alginate have an average size of 15.4 nm with a 5.5-nm standard deviation, Figure 3B. The nanocomplex’s polydispersity index (PDI) was 0.49, indicating medium monodispersity in aqueous solution. Zeta potential of AgNPs changed from −36.7 mV for bare silver nanoparticles to −54.8 mV with alginate due to the presence of carboxylic acid groups in alginates, Figure 2C. The addition of cisplatin neutralized the negatively charged carboxylic acid groups of alginates which resulted in a shift of the zeta potential to −46.3 mV This zeta potential measurements also showed the stability of the nanocomplex’s dispersion (Keshavarz et al., 2018).

Nanoparticle morphology and shape were determined using TEM measurements. TEM micrographs showed that the alginate AgNPs have spherical shapes surrounded by an alginate network and are uniformly distributed without significant agglomeration, Figure 2A. In contrast, the AgNPs have variable shapes with clear agglomeration, Figure 3A.

**FIGURE 4:**
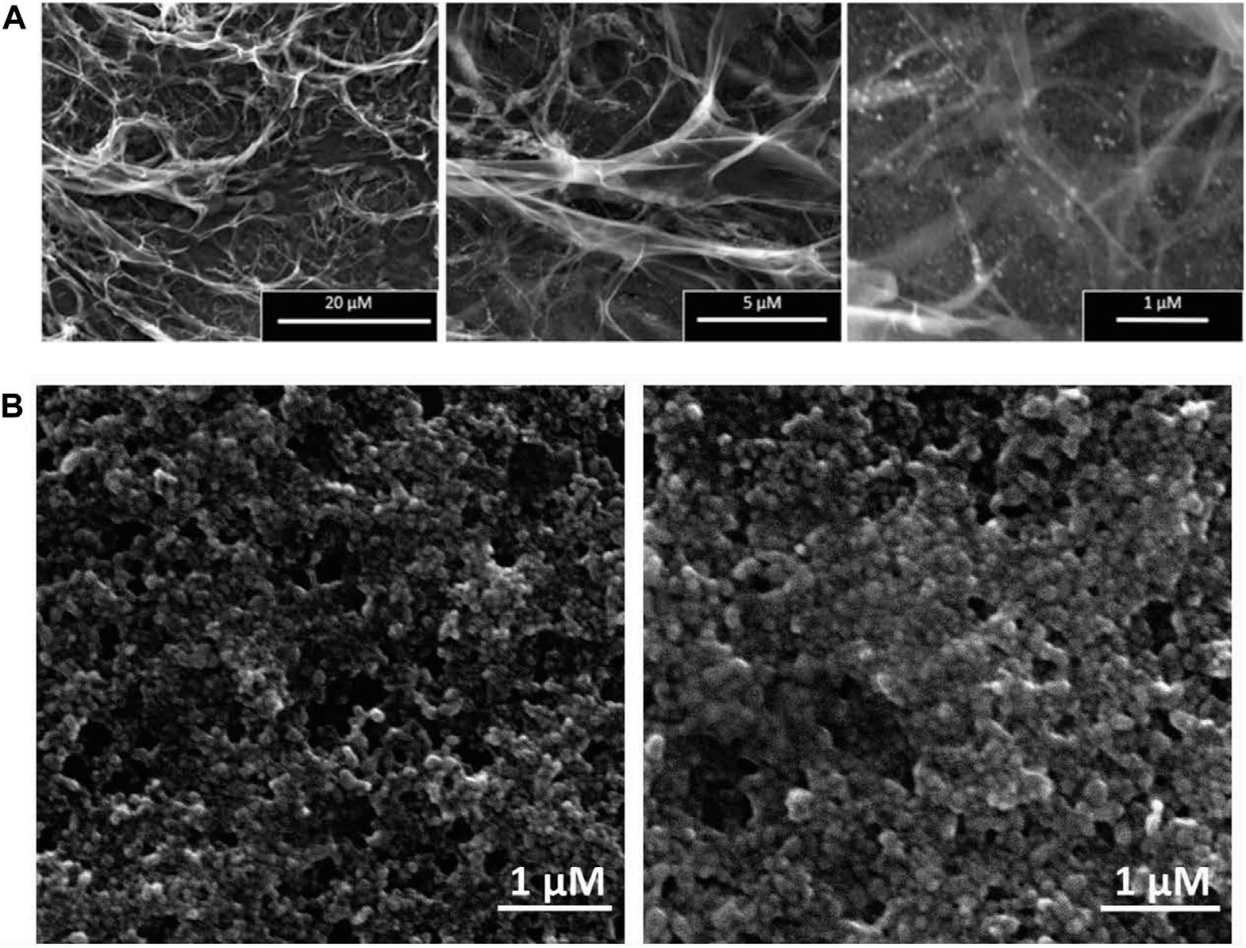
SEM Analysis of Synthesized Nanocomplex and AgNPs: **(A)** The surface morphologies of the synthesized nanocomplex illustrates a smooth outer surface with a slightly porous and lamellar structure. **(B)** demonstrates aggregations of spherical-shaped silver nanoparticles (AgNPs).

**FIGURE 5:**
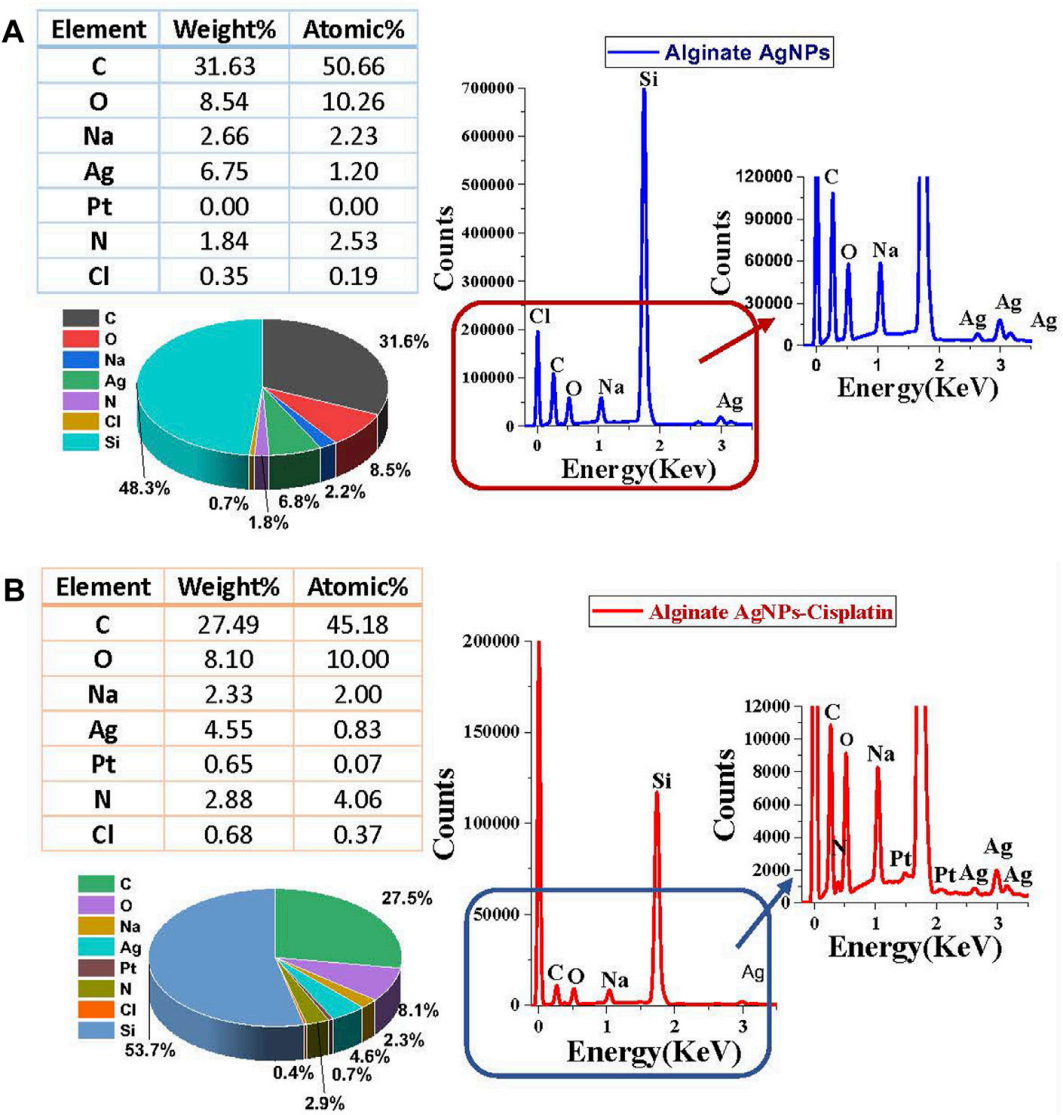
Surface Energy Dispersive X-ray (EDX) spectra of synthesized nanocomplex before and after adding Cisplatin: **(A)** Spectra of Alginate AgNPs. **(B)** Spectra of Alginate AgNPs with a Cisplatin.

**FIGURE 6:**
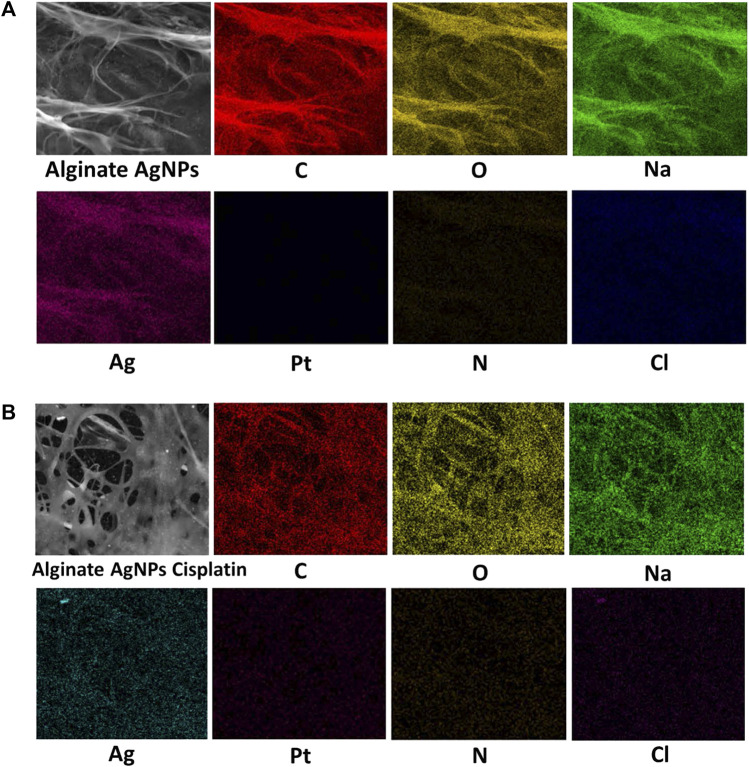
EDX mapping analysis of Alginate AgNPs with and without Cisplatin. **(A)** Alginate AgNPs. **(B)** Nanocomplex Alg-AgNPs-CisPt.

The surface morphologies of the synthesized nanocomplex were determined using SEM. As shown in Figure 4A, the outer surface is smooth and displays a slightly porous and lamellar structure due to the three-dimensional network of alginate blocks. The small AgNPs become shiny and transparent upon magnification. Figure 4B shows the aggregation of spherical silver nanoparticles. Figure 5 depicts a standard EDX spectrum of nanocomplex sample before and after the addition of cisplatin. The peaks at 0.25, 0.5, and 1.1 keV are attributed to elements carbon, oxygen, and sodium of sodium alginate. An intense peak corresponding to silicon is seen at 1.74 keV and is attributed to the silicon substrate used on the mounting base. Distinct peaks between 2 and 4 keV are associated with silver of the AgNPs in the prepared nanocomplex as reported in previous literature (Puchalski et al., 2007; Balavandy et al., 2015; Singh et al., 2015; Pandey and Ramontja, 2016). The presence of cisplatin is confirmed by the appearance of a new nitrogen peak at 0.39 keV and platinum peaks at 1.59, 2.05, and 2.33 keV. In addition, EDX mapping analysis reveals the elemental distribution of carbon, oxygen, sodium, silver, platinum, nitrogen, and chlorine in the surface analysis of nanocomplexes synthesized with and without cisplatin, Figure 6. The surface of the alginate AgNPs shows more carbon and silver elements, but no platinum peaks. However, with the addition of cisplatin, the chlorine, nitrogen, and platinum peaks became more apparent, which clearly suggests the incorporation of cisplatin in alginate/AgNPs nanocomplex.

### 3.2 *In vitro* cytotoxicity of alginate-AgNPs-cisPt using MTT

**FIGURE 7:**
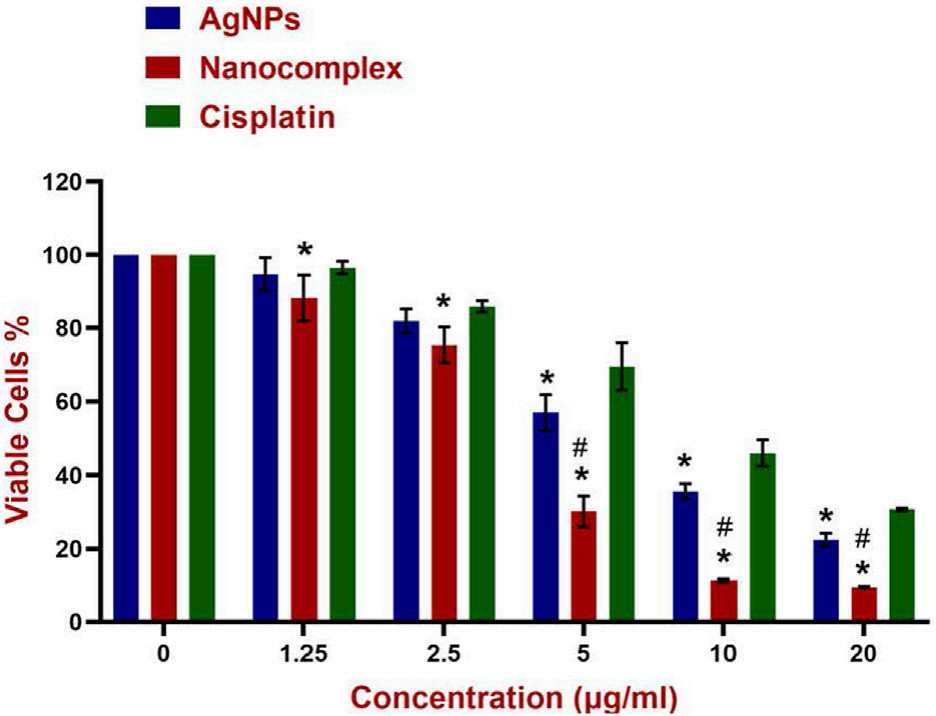
Cytotoxicity of AgNPs, Nanocomplex, and Cisplatin on the MDA-MB-231 cells. Data represents error bars, mean, ±SD, (*n* = 3). The grouped graph shows mean, and SD error bars. Analysis of Variance (ANOVA) test followed by multiple comparison Tukey’s *post hoc* test was used to compare cell viability outcomes between the AgNPS, Nanocomplex, and Cisplatin groups. (In all cases, *p* < 0.05 was considered to indicate a statistically significant difference. * Indicates a significant difference (*p* < 0.02) compared to Cisplatin with the same concentration. # indicates a significant difference (*p* < 0.0001) compared to AgNPs.

**FIGURE 8:**
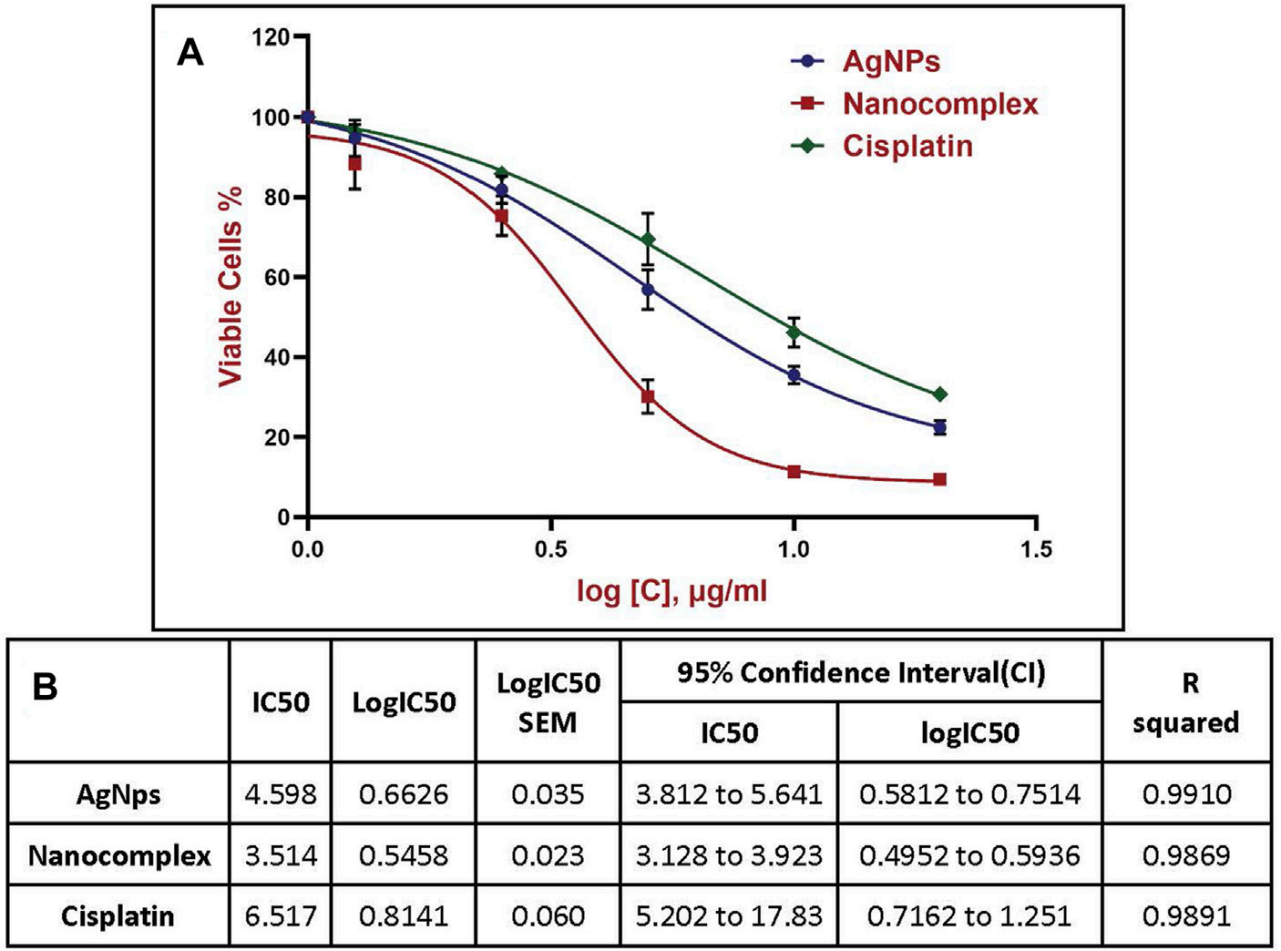
Half-maximal inhibitory concentration (IC50) using nonlinear regression, **(A)** Four-parameter logistic (4 PL) curve fitted to the dose response data. For curve fitting, the concentrations were expressed in a logarithmic scale. **(B)** Data corresponding to the various plots shown in (A).

In this study, the colorimetric change from yellow MTT to purple formazan was used to measure the cell viability. Figure 7 demonstrates the viability of MDA-MB-231 cells treated with AgNPs, alg-AgNPs-CisPt nanocomplex, and cisplatin. As explained in the experimental section, AgNPs and free cisplatin concentrations were similar to those encapsulated in the nanocomplex. As can be seen in Figure 7, at the highest dose of 20 μg/mL, cell viability for AgNPs, nanocomplex, and cisplatin was 22.4%, 9.5%, and 30.5%, respectively. By increasing the concentration of each treatment, AgNPs and cisplatin dramatically decreased cell viability, whereas the nanocomplex produced more cell death than AgNPs and cisplatin at the same drug concentration. The nanocomplex was therefore more effective at killing cells compared to cisplatin or AgNPs alone. We analyzed the IC50 data based on the results of MTT assays. Figure 8 provides dose response data with the corresponding analysis and related IC50 values for the nanocomplex as opposed to its components as standalone treatments. Figure 8 further confirms that the components in the nanocomplex work synergistically to provide a platform that is more effective in killing MDA MB-231 cancer cells.

### 3.3 *In vitro* staining by acridine orange/propidium iodide

**FIGURE 9:**
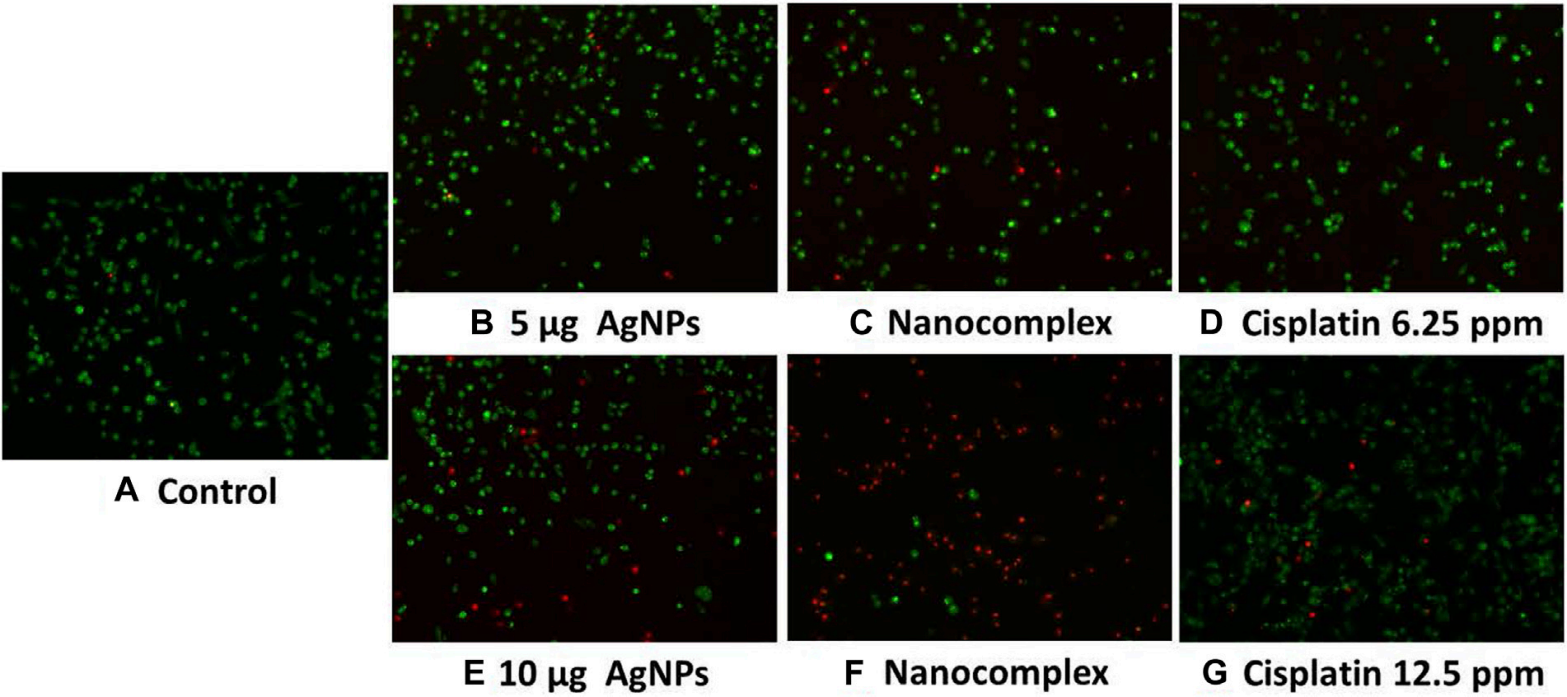
Acridine Orange and Propidium Iodide fluorescent staining of MDA MB-231 cells, **(A)**. Control, **(B–D)** with lower concentration. **(E–G)** with higher concentration.

MDA MB-231 Cells treated with AgNPs, nanocomplex, and cisplatin at higher concentrations (10 ug/mL) exhibit higher cytotoxic rates compared to cells treated at lower concentrations (5 ug/mL), as shown in the fluorescence images, Figure 9. The nanocomplex-treated cells exhibited the highest cytotoxicity compared to those treated with AgNPs or cisplatin separately for the same level of AgNPs and cis-PT loading.

### 3.4 *In vitro* apoptosis/necrosis assay using annexin V-PI assay

**FIGURE 10:**
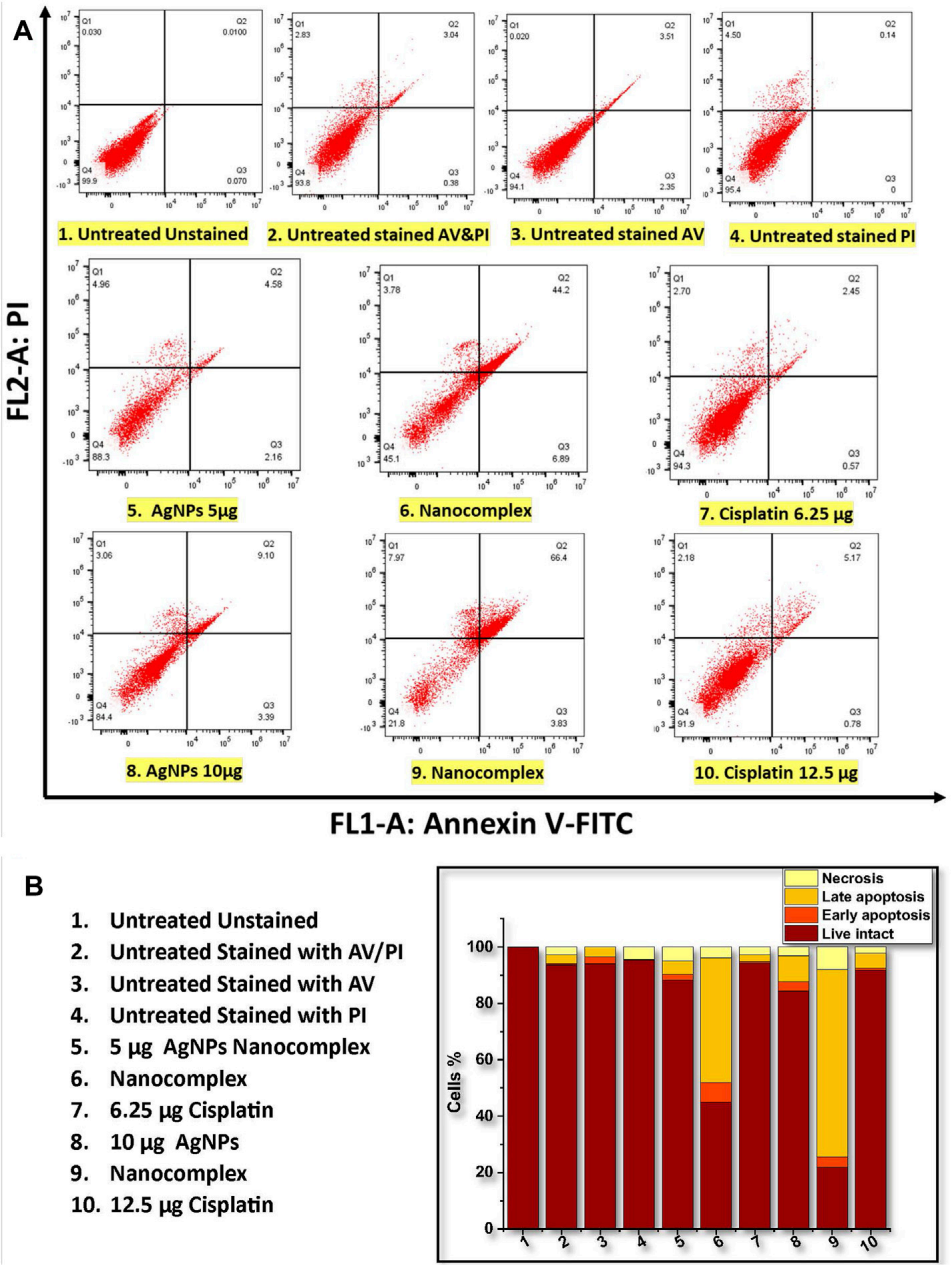
Flow cytometric analysis of annexin V-PI staining of MDA-MB-231 cells. **(A)** Dot plots of untreated and treated MDA-MB-231. **(B)** The population of cells (total of 100%) that are viable, in the early and late apoptosis, and necrosis state.

Figure 10 summarizes the results of the annexin V-PI assay. Figure 10A shows that the proportion of apoptotic cells (Early and late apoptosis) was 6.74%, 51.09%, and 3.02% for 5 μg/mL AgNPs, for the nanocomplex (at 5 μg/mL AgNPs-6.25 μg/mL cisplatin), and for 6.25 μg/mL cisplatin, respectively. Likewise, the higher dose at 10 μg/mL AgNPs, nanocomplex (10 μg/mL AgNPs-12.5 μg/mL cisplatin), and 12.5 μg/mL cisplatin exhibited 12.49%, 70.23%, and 5.95% of apoptotic cells, respectively. Figure 10B shows relative bar graphs summarizing the observations listed above. Based on these findings, exposure to a high concentration (10 μg/mL) of AgNPs, AgNPs-cisplatin, and cisplatin had a significant impact on apoptosis than the low concentration. Most importantly, in both cases, the combined silver nanoparticles and cisplatin in the nanocomplex showed an increased synergistic effect compared to the effect of individual components.

### 3.5 Western blot analysis

**FIGURE 11:**
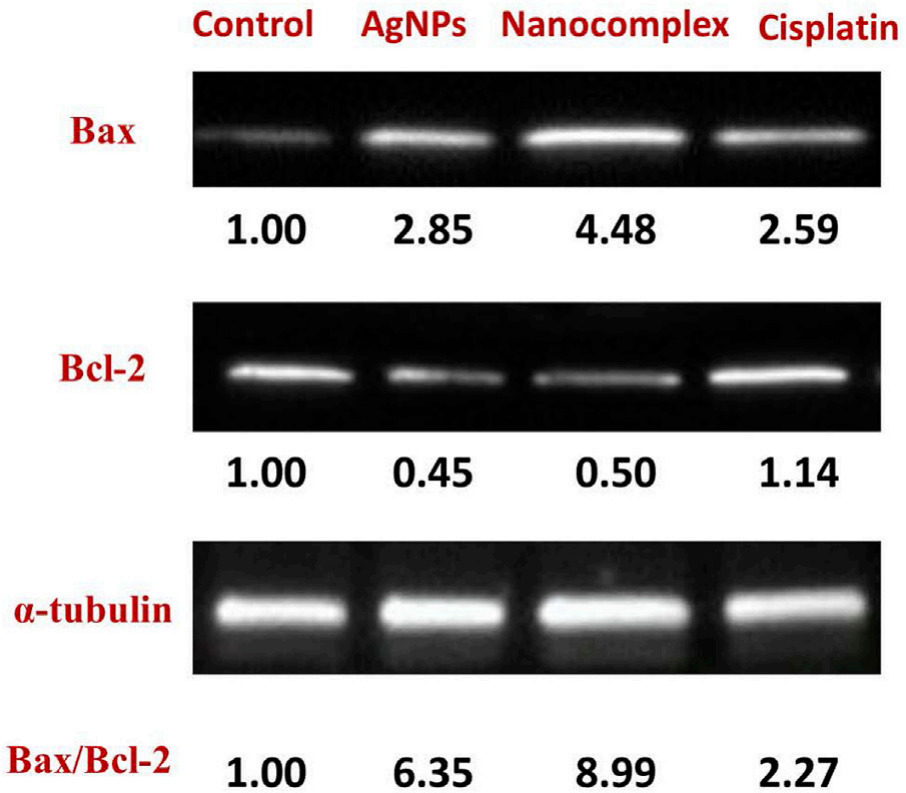
Western blot analysis of proteins related to apoptosis in MDA-MB-231 cells.

The levels of expression of apoptotic and anti-apoptotic proteins were evaluated using Western blot to investigate the mechanism by which AgNPs, nanocomplex, and cisplatin cause cells to undergo apoptosis. As can be seen in Figure 11, treatment of MDA-MB-231 cells with AgNPs, the nanocomplex, and cisplatin, led to a reduction in the amount of the anti-apoptotic protein Bcl-2, while an increase was observed in the level of the pro-apoptotic protein BAX.

### 3.6 ROS detection by 2′,7′ Dichloro dihydro fluorescein diacetate (DCFH-DA) assay using fluorescence microscopy and flow cytometry

In our study, MDA-MB231 cells treated with 5 μg/mL AgNPs, nanocomplex (5 μg/mL AgNPs-6.25 μg/mL cisplatin), and 6.25 μg/mL cisplatin produced 91%, 69.3%, and 95%, respectively, more than untreated cells taken as a control. However, when the cells were treated with higher dose at 10 µg AgNPs, nanocomplex (10 μg/mL AgNPs-12.5 μg/mL cisplatin), and 12.5 μg/mL cisplatin, the percentage of ROS decreased.

## 4 Discussion

AgNPs are extremely cytotoxic to triple-negative breast cancer (TNBC) cells at dosages that are non-cytotoxic to normal breast epithelial cells. As reported by Jessica Swanner et al., AgNPs are mechanically ingested by both cancerous and non-cancerous breast cells, but quickly destroy only cancerous cells. Cancer cells lose their antioxidants and get endoplasmic reticulum stress when exposed to silver nanoparticles, contrary to normal breast epithelial cells. The same literature report showed that systemically administered AgNPs hinder the proliferation of TNBC tumor xenografts in mice at non-toxic doses. The literature reports demonstrated the safety and efficacy of AgNPs as a therapy for TNBC (Swanner et al., 2019). Also, Cale D. Fahrenholtz and colleagues examined the effects of AgNP administration on A2780, SKOV3, and OVCAR3 ovarian cancer cell lines. They showed that AgNPs were extremely toxic to A2780, SKOV3, but far less to OVCAR3. In addition, the treatment of both A2780 and OVCAR3 cells with the mixture of AgNPs and cisplatin showed some enhanced effect of AgNPs and cisplatin in the treatment of ovarian cancer (Fahrenholtz et al., 2017).

In the current study, we developed and validated a functionalized nanocomplex that was formed from alginate hydrogel network coated silver nanoparticles and embedding cisplatin (Alg-AgNPs-CisPt) that has drug modulation and therapeutic efficacy against breast cancer cell line. Polymeric nanoparticles are better than free drugs in a number of ways: it keeps the drug from breaking down too quickly, making it easier for the drug to get into a specific target tissue. It also controls the drug’s delivery and allows a higher dose to get into the cells, protecting the drug from early physiological interference, which lowers its toxicity (Pridgen et al., 2007; Parveen and Sahoo, 2008; Masood, 2016). Different biodegradable polymer nanoparticles have been developed to treat breast cancer over the past few years. Biodegradable polymers, which can be divided into synthetic and natural polymers depending on where they come from, have shown the most promise for making drug delivery systems for anticancer drugs. The biodegradability and biocompatibility of natural polymers make them significantly safer than synthetic polymers. Natural polymers are typically used in the development of cancer-targeted drug delivery systems. One of the most used natural water-soluble linear anionic polysaccharides is sodium alginate (SA). Jing Huang et al. reported the successful fabrication of AgNPs supported by alginate-modified magnetite nanoparticles (Fe_3_O_4_/Alg-Ag NPs). The magnetic Fe_3_O_4_ nanoparticles were coated with alginate and innoculated with AgNPs on the surface. They used alginate as a stabilizing and capping agent for the AgNPs. The Fe_3_O_4_/Alg-Ag nanocomposite exhibited exceptional antioxidant properties against common free radicals. In addition, these nanoparticles exhibited dose-dependent anti-lung cancer activity against NCI-H1975, NCI-H1563, and NCI-H1299 cell lines without cytotoxicity towards the normal cell line (HUVEC) (Huang et al., 2022). Also, Sayuri Gounden et al. synthesized AgNPs, functionalized them with chitosan, and loaded them with cisplatin. These composite nanoparticles induced significant cell death in MCF-7 and SKBR-3 human breast cancer cell lines, as measured by their cytotoxicity profiles compared to free drugs resulting in over 50% cell death. Their findings demonstrate a potentially additive effect of the anti-cancer drug delivery system that targets breast cancer cells selectively (Gounden et al., 2021). Here, we used sodium borohydride as a reductant and sodium alginate as a stabilizing agent to synthesize silver nanoparticles encapsulated in alginate polymer network and embedding cisplatin to form the nanocomplex (Alg-AgNPs-CisPt). The silver nanoparticle dispersions that were prepared using this method were found to be uniform, stable, and with no aggregation over a period of 12 months. The ability of Ag metallic sites to interact with negatively charged carboxylate groups from alginate is thought to have a positive effect on AgNPs formation, and this interaction stabilized the AgNPs in the colloidal suspension of Alg-AgNPs. The carboxylate groups of alginates seem to prevent nanoparticle aggregation through electrostatic repulsion, which improves the colloidal suspension’s stability. Alginate possesses an abundance of carboxylate and hydroxyl groups, which serve as stabilizing functionalities. The presence of these groups on the surface of alginate acts as a capping agent that can form bonds with AgNPs, which further enhances the colloidal stability of the AgNP-alginate sample (Pandey and Ramontja, 2016).

In pharmacology and toxicology, viability tests are essential for explaining the cellular response to a harmful and foreign substance. In addition, they provide information regarding cell survival, death, and metabolic processes (Foldbjerg et al., 2011; Beer et al., 2012; Reddy et al., 2014; Pandian et al., 2015). Here, we used MTT assay (Mukherjee et al., 2012) which assesses mitochondrial activity in living cells to convert a yellow tetrazolium substance into blue formazan crystals as seen in Figure 7. Overall, the nanocomplex was more efficient at inducing cell death than either cisplatin or AgNPs alone. Additional studies such as fluorescence microscopy assays and apoptosis analysis have been conducted to confirm the MTT results. The propidium iodide (PI) and acridine orange (AO) staining allowed us to distinguish between dead and living cells due to binding to nucleic acid as shown in Figure 9. AO enters both dead and living cells and mark live cells with green fluorescence, whereas PI only identifies dead cells with red fluorescence (Krishnasamy et al., 2014; Devanesan et al., 2017). The AO/PI viability assay also showed that the nanocomplex exhibited enhanced toxicity to MDA-MB-231 cells due to synergistic effects of AgNP and cis-Pt as constituents in the nanocomplex. Annexin V-FITC/propidium iodide double staining was used and examined by flow cytometry and the results are presented in Figure 10. Phosphatidylserine (PS) is translocated to the plasma membrane’s outer leaflet in apoptotic cells that have lost membrane phospholipid asymmetry. Other apoptotic processes, which including loss of membrane integrity, DNA shattering, and chromatin condensation, precede PS translocation. Annexin V is a 35–36 kDa calcium-dependent binding protein with high affinity to PS, and the positive detection of Annexin V to translocated PS is an indicator of apoptosis (AshaRani et al., 2009; Wei et al., 2010). On the other hand, propidium iodide is a DNA intercalating agent that enters dead nucleated cells with compromised membranes and generates its known red fluorescence.

Program cell death, or apoptosis, is a genetic program that has been preserved throughout evolution. Cancer alters cellular homeostasis, upsetting the equilibrium between pro-apoptotic and anti-apoptotic proteins. According to variances in cell types and medications, apoptosis mechanisms can be very complex. There are two primary apoptotic processes: Extrinsic pathway (death receptor pathway) and Intrinsic pathway (mitochondrial) (Elmore, 2007). Intrinsic mitochondrial pathway is considered to perform a significant role in the anticancer treatment response. This process is initiated by Bcl-2 family proteins, which are constantly overexpressed in many cancer cells (Ou et al., 2014). These proteins stop the release of cytochrome *c* and prevent programmed cell death, whereas BAX is a member of the Bcl-2 family that initiates apoptosis (Gao and Dou, 2001; Shangary and Johnson, 2002; Autret and Martin, 2009). Bax is mostly found in the cytosol of healthy cells, but when apoptotic signaling begins, it undergoes a structural transformation and becomes more associated to the membrane. Bax increases the permeability of mitochondrial membranes by engaging with ion channel and making pores in the outer mitochondrial membrane. This causes the release of cytochrome *c* as well as other apoptotic components (Ou et al., 2014; Abosharaf et al., 2020), In our work, the treatment of MDA-MB-231 cells with the nanocomplex causes the highest ratio of BAX/Bcl-2 proteins, Figure 11. This suggests that the combination of silver nanoparticles and cisplatin within the same delivery platform results in the highest increase of apoptosis levels as evidenced by the highest level of BAX/Bcl-2 ratio. This is consistent with the flow cytometry findings in Figure 10, which demonstrate an elevation in apoptotic cell population.

**FIGURE 12:**
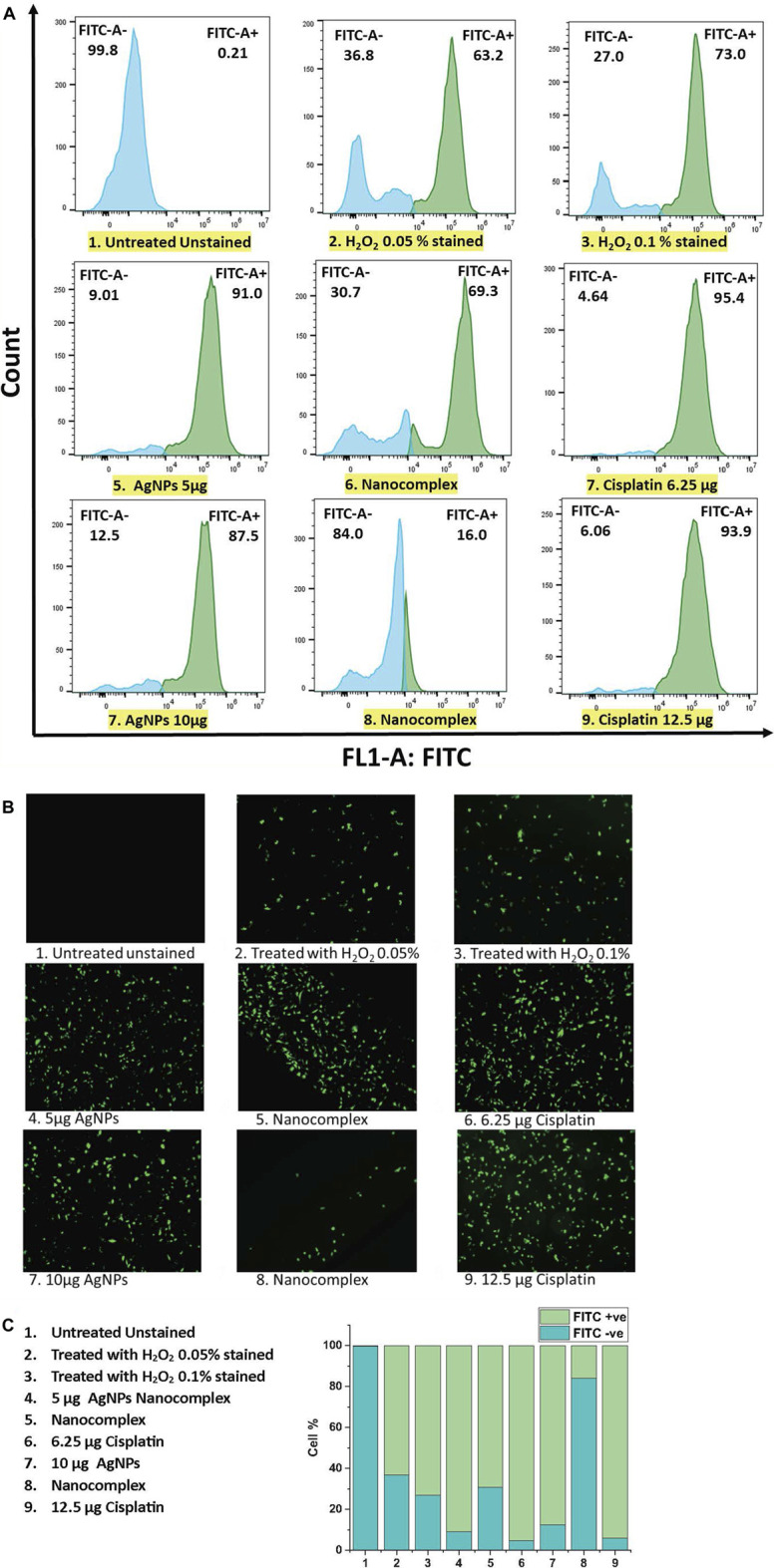
**(A)** ROS detection by Flow cytometric analysis **(B)** ROS generation by fluorescence microscopy analysis. **(C)** The population of cells (total of 100%) that are FITC + ve and FITC -ve using flow cytometry.

In general, cells treated with foreign chemicals, like anti-cancer medications, nanoparticles, and cytotoxic agents, are constantly exposed to the damaging impact of highly reactive oxidizing molecules, which ultimately results in cell structure destruction. Reactive Oxygen Species (ROS) has been considered a crucial activator of cell apoptosis. ROS are generated by healthy cells as well and play a crucial role in several physiological processes. Under excessive levels of reactive oxygen species, a cascade of biological reactions culminates in the damage of DNA, oxidative degradation of lipid, cell cycle halt, and apoptosis. Figure 12 illustrates the results of flow cytometry and fluorescence microscopy with DCFH-DA to measure the intracellular ROS level in MD-MB-231 cells exposed to AgNPs, to the synthesized nanocomplex, and to cisplatin. The DCFH-DA probe detects intracellular oxidative stress after esterase-enabled cell access (Kalyanaraman et al., 2012; Han et al., 2017; Delalat et al., 2022). The two-electron oxidation of DCFH inside the cell yields the highly fluorescent dichlorofluorescein (DCF), which can be monitored using various fluorescence-based techniques such as flow cytometry and fluorescence microscopy (Juarez-Moreno et al., 2017). The higher-dose treatment with the nanocomplex seems to generate a lower level of ROS signal. However, the signal decrease measured by flow cytometry is simply the result of cell detachment, which is expected to decrease the prevalence of attached fluorescent cells, Figure 12A. In fact, this interpretation is confirmed by fluorescence microscopy, Figure 12B, which shows less attached cells when the sample is treaded with the high dose nanocomplex compared to the low dose treatment.

## 5 Conclusion

Alginate-functionalized AgNPs nano-delivery system successfully encapsulated cisplatin and exhibited favorable characteristics including small size, monodispersity, and absence of agglomeration. We have described the preparation of alginate functionalized AgNPs with and without cisplatin as an active drug nanoplatform. We have conducted detailed analysis of the prepared nanocomplex. In addition, we showed that this system exhibits cytotoxicity for MDA-MB-231 breast cancer cells, with over 90% cell death as confirmed by apoptosis assays and staining. Most importantly, we show that the nanocomplex exhibits a synergistic toxic effect of the silver nanoparticles and cisplatin towards MDA-MB-231 breast cancer cells by enhancing the preponderance of apoptotic signals and thus cell death. The hydrogel-coated silver nanoparticle platform embedding cisplatin has the potential to use less amount of cisplatin drug for enhanced efficiency against cancer cells and therefore opens the road for a treatment regimen with relatively less side effects.

## Data Availability

The original contributions presented in the study are included in the article/Supplementary Material, further inquiries can be directed to the corresponding author.
